# Structure and Performance of All-Green Electrospun PHB-Based Membrane Fibrous Biomaterials Modified with Hemin

**DOI:** 10.3390/membranes13050478

**Published:** 2023-04-28

**Authors:** Polina M. Tyubaeva, Ivetta A. Varyan, Alexey V. Krivandin, Olga V. Shatalova, Anatoly A. Olkhov, Anatoly A. Popov, Huaizhong Xu, Olga V. Arzhakova

**Affiliations:** 1 Academic Department of Innovational Materials and Technologies Chemistry, Plekhanov Russian University of Economics, 36 Stremyanny per., Moscow 117997, Russia; polina-tyubaeva@yandex.ru (P.M.T.);; 2 Emanuel Institute of Biochemical Physics, Russian Academy of Sciences, 4 ul. Kosygina, Moscow 119334, Russia; 3 Department of Biobased Materials Science, Kyoto Institute of Technology, Kyoto 606-8585, Japan; 4 Faculty of Chemistry, Lomonosov Moscow State University, Leninskie Gory 1/3, Moscow 119991, Russia

**Keywords:** PHB, Hemin, surface modification, membrane functionalization, fibrous nonwoven membranes, supramolecular structure, permeability

## Abstract

This work addresses the challenges concerning the development of “all-green” high-performance biodegradable membrane materials based on poly-3-hydroxybutyrate (PHB) and a natural biocompatible functional additive, iron-containing porphyrin, Hemin (Hmi) via modification and surface functionalization. A new facile and versatile approach based on electrospinning (ES) is advanced when modification of the PHB membranes is performed by the addition of low concentrations of Hmi (from 1 to 5 wt.%). Structure and performance of the resultant {HB/Hmi membranes were studied by diverse physicochemical methods, including differential scanning calorimetry, X-ray analysis, scanning electron microscopy, etc. Modification of the PHB fibrous membranes with Hmi allows control over their quality, supramolecular structure, morphology, and surface wettability. As a result of this modification, air and liquid permeability of the modified electrospun materials markedly increases. The proposed approach provides preparation of high-performance all-green membranes with tailored structure and performance for diverse practical applications, including wound healing, comfort textiles, facial protective masks, tissue engineering, water and air purification, etc.

## 1. Introduction

Electrospinning (ES) is credited to be a cutting-edge strategy for the preparation of high-performance membrane materials with excellent properties [1,2,3]. This technology offers substantial advantages for structural design of non-woven fibrous membrane materials with task-oriented properties for diverse practical applications, including water recovery, desalination, biomedical purposes, comfort textiles, oil-water separation, protective clothing, tissue engineering, etc. [3,4,5]. This mainstream direction of membrane science and technology is facing numerous challenges, including control over structure, morphology, overall porosity, pore size, mechanical properties, hydrophilic-lipophilic balance (HLB), biocompatibility, performance of fibrous membrane materials in specific applications, and, finally, the development of optimal protocols of membrane preparation and transition to large-scale production level [1,6].

The benefits of electrospun fibrous membranes are primarily related to their high porosity, interconnected porous structure, good pore channel connectivity, controlled pore size, high surface area, a high surface area-to-pore volume ratio, and uniform pore distribution [3,6]. Recent studies in this direction focused on the improvement in their performance, thus broadening the scope of their practical applications as membranes and filters. This objective can be achieved in two ways: optimization of the ES protocol and use of new polymers (I) or by functionalization and modification of existing nonwoven fibrous membranes. The performance of electrospun membranes can be optimized by understanding the whole chain of their fabrication starting with proper choice of polymers and modifying additives, selection of optimal feed solutions, control over the ES process via variations in operating parameters, etc. [6]. This complex approach can provide the controlled design of task-oriented nonwoven membrane materials with desired morphology and performance. Morphological and topological features of nonwoven membrane materials can also be improved using various methodologies, including molecular bonding, in situ polymerization, and addition of diverse molecular dopants. Surface modification of ES nonwoven membranes can be performed by nanoparticle coating, treatment with chemicals or heat, grafting, and interfacial polymerization [7,8,9,10,11].

One of the important challenges in the preparation of nonwoven membrane materials is concerned with the development of all-green biodegradable and biocompatible materials with high performance which can be used for special purposes (tissue engineering, wound healing, implantation, orthodontics, etc.) and easily utilized [12,13,14,15,16,17,18]. However, there are strict limitations and demands for the choice of polymers and additives for the preparation of biomedical membrane materials [19,20]. The best candidates for this purpose are biocompatible polymers of natural origin and natural additives which proved their efficacy in biomedicine [21], wound healing [22], creation of implants [23], matrices [24], and cell scaffolds [25]. Among biocompatible and biodegradable polymers, collagen, fibroin [26], gelatin [27], chitin [28], polycaprolactone [29], polyhydroxybutyrate [30] seem to be the advantageous choice due to the optimal structure/property combination (mechanical strength, physicochemical properties, biocompatibility, cost efficacy, etc.). Of special interest is a natural biodegradable polyester, poly-3-hydroxybutyrate (PHB) which is synthesized from renewable natural sources. This polymer with good service properties (for example, high thermal stability over a broad temperature interval up to 150 °C) is biodegradable, biocompatible with a human body, and can be sterilized by various physical and chemical methods [31,32]. PHB can serve as a perfect polymer host matrix for further modification [33,34] and development of efficient biomedical membranes [35,36]. Moreover, due to its good biodegradability, the problems of further utilization of the PHB-based materials can be easily solved. Of special importance is the selection of efficient modifying additives for PHB, which, on one hand, can improve the efficiency of ES process and, on the other hand, cam impart new functionalities to the nonwoven membranes such as antimicrobial activity, hydrophilicity and hydrophilic-lipophilic balance (HLB), strength, etc.

Iron-containing porphyrin Hemin (Hmi) with a coordination number 5 seems to be a promising candidate [37], as this compound is endogenously produced in the human body, biocompatible with living organisms, and can be used for the biomedical applications [38,39]. Hemin is characterized by high thermal stability [40] and antimicrobial activity against pathogenic organisms [41]. Moreover, the advantages of Hmi involve an easy and reliable synthesis from natural sources. Hence, in this work, this natural additive was selected for the modification of the PHB fibrous materials and preparation of high-performance membranes with task-oriented functional properties.

This work offers a facile and versatile approach for the modification and functionalization of PHB-based nonwoven fibrous membranes by a natural functional additive Hemin by electrospinning and highlights the benefits of this approach for the preparation of all-green PHB/Hmi membrane materials with high performance and good mechanical characteristics for diverse practical applications.

## 2. Materials and Methods

### 2.1. Materials

In this work, biodegradable polymer, poly-3-hydroxybutyrate (PHB) (16F series, BIOMER, Schwalbach am Taunus, Germany) was used. Molecular weight of PHB was 206 kDa; powder density was 1.248 g/cm^3^, degree of crystallinity of the PHB powder was 59%. In this work, we used Hemin (Hmi) from the bovine blood (Aldrich Sigma, Saint Louis, MO, USA). Phosphate-buffered saline (PBS) (Biolot, St. Petersburg, Russia), chloroform (Biolot, St. Petersburg, Russia), N,N-dimethylformamide (Biolot, St. Petersburg, Russia) were used as solvents.

### 2.2. Methods

#### 2.2.1. Preparation of the PHB/Hmi Feed Solutions

For electrospinning, feed solutions of PHB/Hmi with different concentrations of Hmi (1, 3, 5 wt.%) were prepared. Standard volume of the solution was 25 mL. PHB powder was dissolved in chloroform at a temperature of 60 °C under vigorous stirring using an JFS-550S/750S/1100S automated dispersant (Yunlin Li, Beijing, China). Hmi powder was dissolved in N,N-dimethylformamide at a temperature of 25 °C. The solutions were homogenized and used within 12 h after preparation.

Viscosity of the PHB/Hmi feed solutions was measured using a DV2T Metek viscometer (Brookfield, Middleboro, MA, USA) according to the standard procedure (600 mL Griffin beaker; an LV-62 standard spindle) at 25 °C. Electrical conductivity of the PHB/Hmi feed solutions was measured using an S47K conductometer (Mettler Toledo, Albstadt, Germany) at 25 °C.

#### 2.2.2. Preparation of the PHB/Hmi Electrospun Membranes

Electrospun materials were prepared on an EFV-1 unit (IBCHP RAS, Moscow, Russia). Electrospinning (ES) was performed using a single-capillary molding flask. The diameter of a capillary was 0.1 mm, voltage on both electrodes was 17–20 kV, distance between the electrodes was 190–200 mm, gas pressure on the solution was 10–14 kg(f)/cm^2^. 25 mL of PHB solution contains 1.75 g of polymer (7% wt.%); 1 wt.% solution–0.017 g of Hmi; 3 wt.%–0.054 g; 5 wt.%–0.092 g. Hemin was dissolved in 3 mL of the solvent. The solutions were homogenized using an automatic mixer for 30 min.

#### 2.2.3. Scanning Electron Microscopy (SEM)

Surface morphology of the PHB membrane materials was studied on a Tescan VEGA3 microscope (Tescan, Wurttemberg, Czech Republic). Prior to tests, surface of the test samples was decorated with a thin conducting platinum layer.

#### 2.2.4. Analysis of Morphology of the PHB/Hmi Membranes

Average diameter of the electrospun fibers was estimated by the image analysis of the corresponding micrographs using the BX43Olympus Stream Basic software (Olympus, Japan, Tokyo).

#### 2.2.5. Surface Density

Surface density of the test samples was estimated gravimetrically using a Balance XPR106DUHQ/A analytical weighing-machine (Mettler Toledo, Columbus, OH, USA). Surface density, *δ*, g/cm^3^, was calculated as:

(1) $$\delta =\frac{m}{l\times B\times b}$$

where *m* is the weight of the sample; *l* is the length; *B* is the width; *b* is the thickness. The average value was estimated from 10 to 12 measurements. Experimental error was below 3–5%.

#### 2.2.6. Mechanical Properties

Mechanical properties (including strength and elongation at break) were studied using a DVT GP UG 5 testing machine (Devotrans, Istanbul, Turkey). The strain rate was 25 mm/min (without preloading). Strength F_max_(N) was registered automatically. The average values were calculated from, at least, 5 to 7 measurements. Elongation at break *ε* was calculated as:

(2) $$\varepsilon =\frac{\Delta l}{l_{0}}\times 100\%$$

where Δ*l* is the difference between the final and initial length of the sample; *l*_0_ is the initial length of the sample. The average statistical error was ±0.2%.

#### 2.2.7. X-ray Diffraction (XRD) Analysis

Structural morphology of the crystalline phase, degree of crystallinity of the test PHB samples and average size of crystallites were estimated by X-ray diffraction analysis on an HZG4 diffractometer (Freiberger Präzisionsmechanik, Freiberg, Germany). The degree of crystallinity was calculated using the standard procedure [42]. Average size of PHB crystallites, *L*_020_, was calculated from the corresponding diffractograms according to the Bragg–Brentano approach using the Selyakov–Scherrer formula [43].

#### 2.2.8. Differential Scanning Calorimetry (DSC)

Enthalpy of fusion and melting temperature were estimated from the DSC tests on a DSC 214 Polyma thermal analyzer (Netzsch, Selb, Germany). A testing procedure involved two heating (from 20 °C to 220 °C) and two cooling runs (from 220 °C to 20 °C). The samples were tested in an argon atmosphere; heating rate was 10 °C/min; cooling rate was 10 °C/min; the weight of the samples was 6–7 mg.

Enthalpy of fusion and melting temperature were automatically recorded by a NETZSCH Proteus software (Netzsch, Selb, Germany) according to the standard technique.

#### 2.2.9. Wettability

Contact angle (CA) serves as a quantitative measure of surface wettability of the PHB/Hmi samples. The measurements were performed using a sessile drop method. Water droplets (2 µL) were applied onto three different surface areas of the nonwoven material by an automatic dispenser. The snapshots were collected using an M9 No. 63649, lens FMA050 optical microscope (AmScope, Moscow, Russia) and analyzed using the Altami studio 3.4 Software. Relative experimental error was ±0.5%.

#### 2.2.10. Air Permeability

Air permeability of the PHB/Hmi membranes as the Gurley number was measured according to the standard protocol [44]. The pressure was 1.22 MPa, volume of the air was 100 mL, the test-area was 6.5 cm^2^. Relative experimental error was ± 5%.

#### 2.2.11. Pressure-Driven Liquid Permeability

Water and phosphate-buffered saline (PBS) permeability of the PHB–Hmi membranes was measured according to the standard procedure [45]. The pressure was 1.22 MPa, volume of the liquid was 10 mL, the test surface area was 6.5 cm^2^. Relative experimental error was ±5%.

#### 2.2.12. Dynamic Vapor Sorption Method

Water vapor sorption analysis was performed using a DVS Advantage (Surface Measurement System; Middlesex, UK) automated moisture sorption instrument at 25 °C. The weight of the samples was 10–14 mg. For correct tests, the samples were initially dried for 600 min under vacuum to remove all water traces. Then, the pressure was supplied to the system (typical partial pressure increase profile: 0–90%).

#### 2.2.13. Fourier Transform IR-Spectroscopy

FTIR spectra of samples of the material were collected on a Lumos BRUKER instrument (Karlsruhe, Germany) by the method of multiple disturbed total internal reflection on a diamond crystal. The resolution was 2 cm^−1^. Measurements were carried out in the range from 600 to 4000 cm^−1^.

## 3. Results and Discussion

Structure, morphology, properties, and performance of all membrane materials are known to be strongly controlled by the method of their preparation. Electrospinning (ES) is credited as a powerful and efficient method for the preparation of a highly porous fibrous structure with a high content of open pores, uniform pore size distribution, and high pore channel connectivity, thus allowing the development of high-performance fibrous membrane materials and filters [46]. The ES approach involves the formation of long thin polymer fibers which are organized into a randomly arranged weblike network structure under the action of physical forces: Coulomb forces, electric field, viscoelastic and surface tension [47].

The quality of the resultant fibrous membranes is strongly governed by the composition of the feed polymer solution. For example, the diameter of thin fibers in the nonwoven weblike membranes defects as glues and spindles between fibers, uniform organization and density of the final fibrous materials critically depend on the viscosity [48], electrical conductivity [49], and composition of the feed polymer solutions as well as on curing conditions: solidification rate and rate of subsequent solvent evaporation [50]. The preparation of high-performance membrane materials demands the search for an optimal balance between polymer/solvent/additive composition of in the feed solution [51]. This work addresses the modification and functionalization of the electrospun PHB-based membrane materials by Hmi and the selection of the proper composition of the feed solutions with sufficient viscosity and electrical conductivity for the preparation of the PHB/Hmi composites with required performance as membrane materials and filters. Information of the composition of the feed solutions and morphology of the PHB-Hmi membrane materials prepared by ES process are listed in Table 1.

Introduction of Hmi into the polymer feed solution was shown to increase its electrical conductivity by 10–40% and viscosity increased by 40–90%. As a result, the average diameter of fibers in the non-woven material decreased by a factor of 2, and overall density was reduced from 0.30 down to 0.17 g/cm^3^ as the content of Hmi in the PHB/Hmi membranes increased from 1 wt.% to 5 wt.%. With increasing the content of Hmi, electrical conductivity of the feed solution was enhanced, and this increase can be explained by the presence of a metal atom in Hemin. This key parameter has a crucial effect on the movement of a polymer solution through a jet upon ES [52], thus allowing a better organization of fibers into a weblike network and providing a uniform thickness of fibers due to the presence of electrically conductive particles [53]. Viscosity of the feed solution depends on the composition of two solvents, and this strategy makes it possible to control the efficiency of the ES process [54]. As a result, optimal selection of the composition of the polymer/additive/solvent feed solutions allows a tailored design of nonwoven membrane materials with controlled structure.

**Figure 1:**
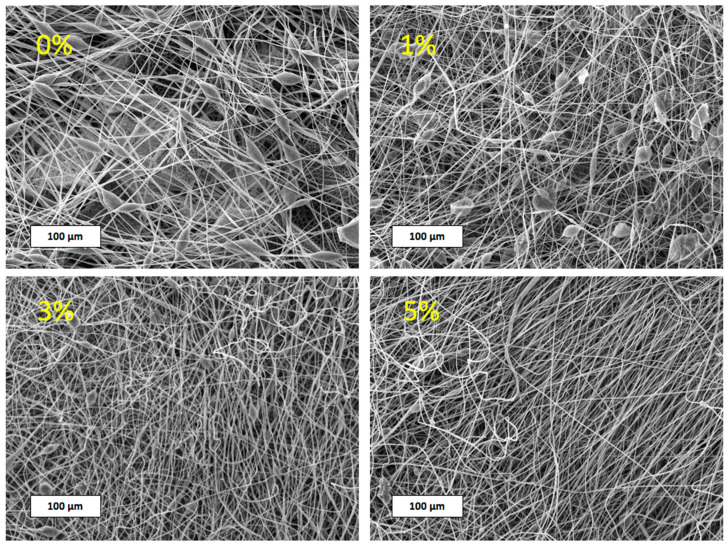
SEM images of electrospun PHB/Hmi fibrous membrane materials with different content of Hemin (from 0 to 5 wt.%).

Figure 1 shows the SEM images of the prepared PHB/Hmi membranes with different content of Hmi (from 0% to 5 wt.%)

The corresponding SEM images (Figure 1) show that the addition of Hmi exerts a marked effect on the morphology of the resultant membrane materials. As the concentration of Hmi increased from 0% to 5 wt.%, the content of structural defects in the nonwoven materials was markedly reduced. The pristine PHB nonwoven membranes contained numerous structural defects seen as glues and spindles. The dimensions of structural defects varied from 4 to 20 µm; the dimensions of glues and spindles—from 80 to 170 µm. The introduction of Hmi even in low concentrations (1 wt.%) lead to a certain improvement in the morphology of the resultant membrane materials: the overall number of all defects decreased, dimensions of structural defects were reduced down to 3–15 µm and the glues became appreciably smaller (50–100 µm). As the content of Hmi increased to 3 and 5 wt.%, dimensions of structural defects decreased down to 2–7 µm. Of special importance was the high quality of fact that the nonwoven membranes containing 5 wt.% of Hmi as they contained no glues and spindles. Hence, the addition of the modifying Hmi agent to PHB nonwovens offers a powerful tool for the control over the quality and morphology of the fibrous membrane materials and provides better operating conditions for ES process.

Modification of the membrane materials by the addition of Hmi via electrospinning also makes it possible to improve the supramolecular structure of the PHB/Hmi composite materials: controlled ordering of both crystalline and amorphous phases and predominant orientation along the fiber axis [55]. In this case, Hmi inclusions in the PHB/Hmi composite served as crystallization sites upon solidification, and the whole structure of the crystalline phase was modified (Table 2). Upon introduction of Hmi to PHB, parameters of orthorhombic crystal lattice of PHB remained unchanged (a = 0.576 nm, b = 1.320 nm, c = 0.596 nm, space group symmetry of *P*2_1_2_1_2_1_) [56]. However, the introduction of Hmi provided a better organization and ordering of crystallites within the crystalline phase of the PHB/Hmi composite. Information on the supramolecular structure of the fibrous PHB/Hmi membrane materials is presented in Table 2.

**Figure 2:**
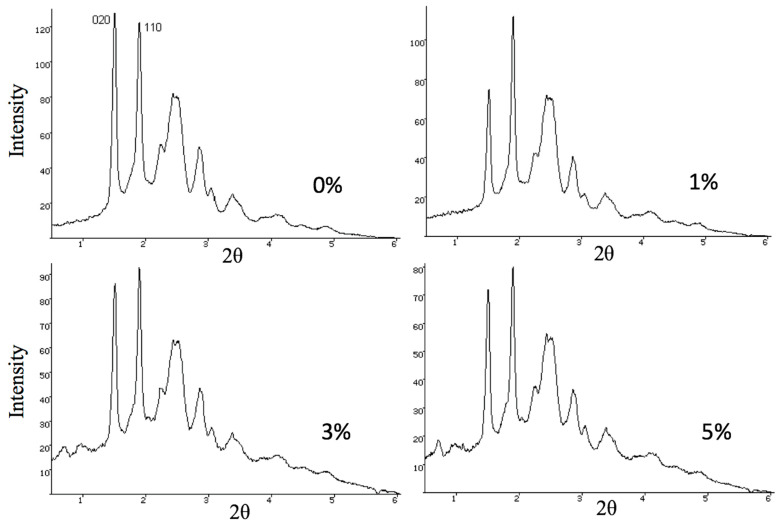
X-ray diffractograms of the electrospun PHB/Hmi materials.

As follows from Table 2, as the content of Hmi in the PHB/Hmi composite membrane materials increased from 0% to 5 wt.%, the degree of crystallinity decreased from 48% down to 41%; the dimensions of crystallites appeared to be slightly increased. It is noteworthy that, upon the addition of 1 wt.% of Hmi, the size of the crystallites increased by ≈23%, whereas the addition of 3% and 5% provided a less pronounced increase (by 16 and 14%, respectively). This tendency can be explained by the aggregation of Hmi in the feed solutions as the concentration of Hmi increased [57]. This assumption also agrees with the X-ray diffractograms of the PHB/Hmi samples (Figure 2).

Comparative analysis of X-ray diffraction patterns of PHB/Hmi (Figure 2) and PHB powder revealed predominant orientation of the PHB crystallites when the content of Hmi was 1 wt.%. Diffractograms of the PHB/Hmi samples containing 3 and 5 wt.% of Hemin (Figure 2) showed weak diffraction maxima at S ≈ 0.71 nm^–1^ (2θ = 6.28°) and S ≈ 0.96 nm^–1^ (2θ = 8.83°), which are not typical of the crystalline structure of PHB or Hmi. This fact, possibly indicates the formation of “hybrid” mixed ordered structures of PHB and Hmi, and this result is consistent with the DSC studies.

**Figure 3:**
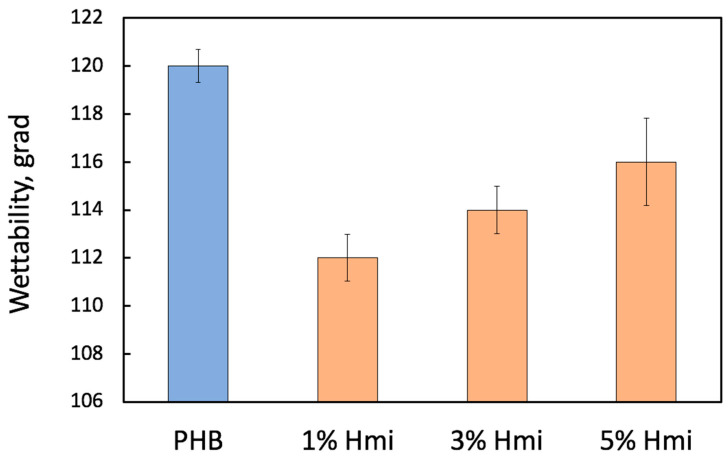
Wettability (or contact angle) of the electrospun PHB/Hmi membrane materials with different content of Hmi (blue—without an additive, orange—with different content of Hmi).

Modification of the PHB nonwoven membranes with Hmi also addresses serious challenges related to their high hydrophobicity which limits the scope of their practical applications [58]. The introduction of Hmi to PHB makes it possible to control the hydrophilic-lipophilic balance (HLB) of the PHB/Hmi membrane materials and to improve their interaction with the body’s liquid media. Figure 3 shows the diagram illustrating the effect of Hmi on the contact angle (CA) of the resultant materials, which serves a quantitative measure of wettability [59,60].

**Figure 4:**
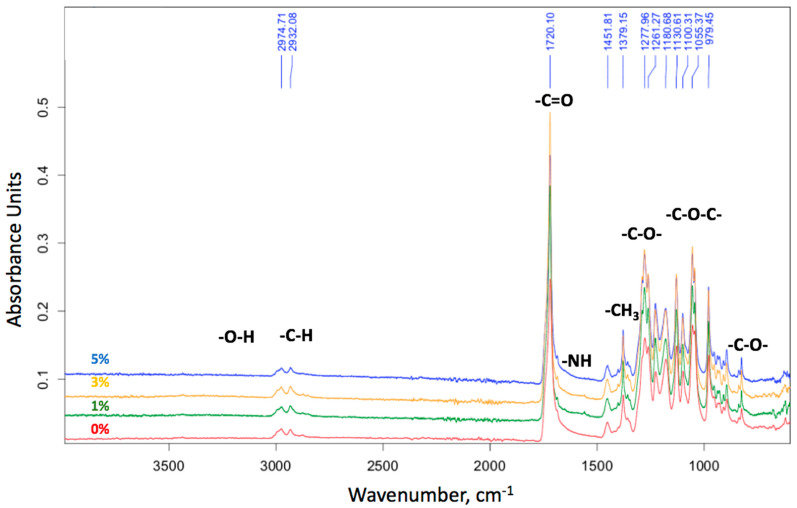
FTIR spectra of the electrospun PHB/Hmi membrane materials.

Figure 3 shows that upon introduction of Hmi, CA decreased, thus indicating an improved wettability of the PHB/Hmi materials. By itself, Hemin is known to be hydrophobic [61]. To understand the chemical composition of the modified PHB/Hmi materials, it seems interesting to analyze the FTIR spectra of the composites. Figure 4 shows the corresponding FTIR spectra of the PHB/Hmi membranes with different content of Hmi.

As follows from Figure 4, the introduction of Hmi to the PHB/Hmi composites provides slight changes in the chemical structure of PHB, including -C=O carbonyl at 1720 cm^−1^, -C-O-C- ester linkage at 1150–1300 cm^−1^, -C-H bonds in methyl radical at 2900–3100 cm^−1^, -CH_3_ at 1380 cm^−1^ [62], or characteristic bands of PHB at 1165–1280 cm^−1^ [63]. However, evident changes were observed for nitrogen-containing groups at 1590–1650 cm^−1^ due to the introduction of low concentrations of Hmi containing nitrogen atoms. When Hmi is bounded or aggregated within the PHB/Hmi sample, its nitrogen atoms and polar -COOH groups in the tetrapyrrole ring contribute to the hydrophilization of the membrane surface.

**Figure 5:**
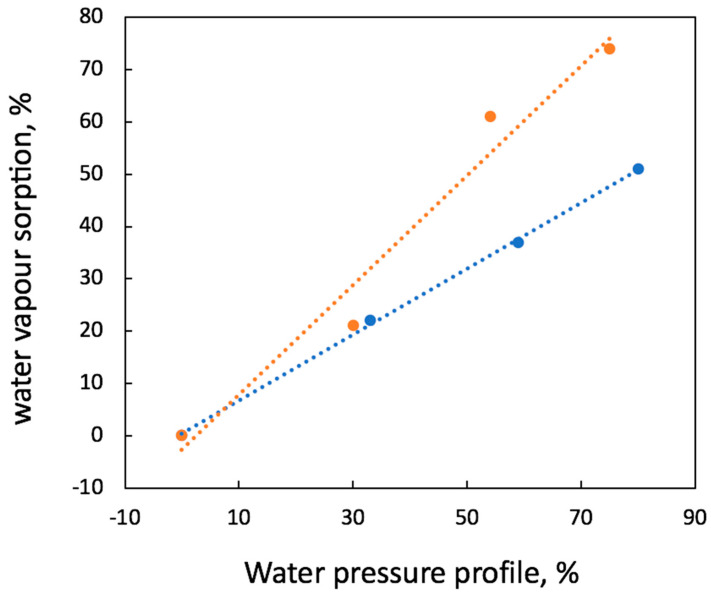
Water uptake versus water pressure: initial PHB (blue) and PHB with 5 wt.% of Hmi (orange).

The performance of the PHB/Hmi membrane materials can be also assessed by their sorption of water vapors. Figure 5 shows the water uptake (or water sorption) plotted against the water vapor pressure for the PHB/Hmi membranes containing 5 wt.% of Hmi.

As follows from Figure 5, water uptake of both PHB and PHB/Hmi materials gradually increased with increasing the water vapor pressure in the closed cell. Both dependences were seen to be nearly linear, thus indicating the stability of the membranes towards the action of water vapors. It is noteworthy that water uptake of the modified PHB/Hmi membrane materials appeared to be higher than that of the pristine PHB. This behavior can be explained by the data on the contact angles when, as the content of Hmi in the composite increased, CA decreased, thus indicating improved wettability. Hence, the addition of Hmi to PHB provides its hydrophilization, and the level of HLB can be controlled by the content of Hmi. This improved wettability can also contribute to enhanced biodegradability of the resultant composite materials, allowing better accessibility of the internal structure of membranes to the bacterial attack.

**Figure 6:**
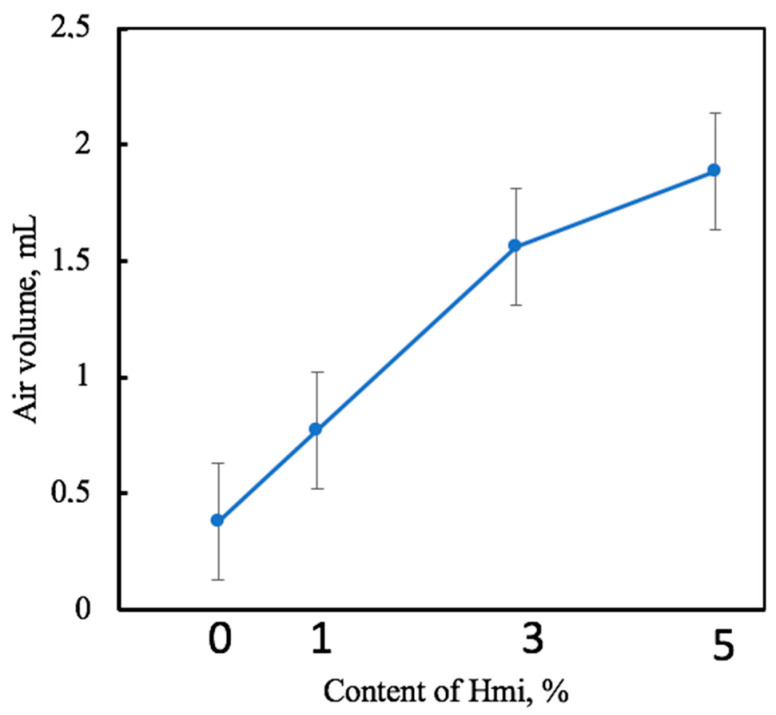
Air permeability of the electrospun PHB/Hmi membrane materials.

Performance of the PHB/Hmi materials as membranes can be estimated by traditional membrane characteristics as air and liquid permeability [57]. Figure 6 presents the data on air permeability of the PHB/Hmi membrane materials with different content of Hmi.

As follows from Figure 6, as the content of Hmi in the PHB/Hmi materials increased, air permeability of the electrospun PHB/Hmi membranes markedly increased from 0.4 to 2.0 mL. The improved air permeability or, in other words, breathability of the PHB/Hmi membranes can be explained by the fact that modification of PHB with Hemin at the stage of electrospinning provides the formation of a more uniform and ordered structure of the filter materials and development of numerous open interconnected pores. Hence, the introduction of Hmi, even in low concentrations, makes it possible to control both HLB and breathability of the PHB/Hmi membranes [64,65].

**Figure 7:**
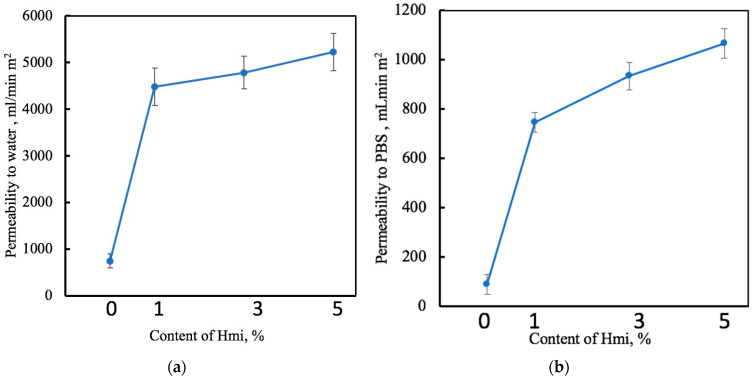
Permeability to water (**a**) and to PBS (**b**) of the electrospun PHB/Hmi membrane materials with different content of Hemin.

The performance of the membrane materials is also evaluated by their permeability to liquids [45], and this parameter is important for their use as membranes and filters in biomedical applications [66]. Figure 7 shows the effect of Hmi on the liquid permeability of the electrospun PHB/Hmi materials for both water and PBS.

As follows from Figure 7, the introduction of Hmi to the PHB/Hmi membranes had a marked effect on their liquid permeability. Water permeability increased by a factor of 6–7, and this fact well agrees with the data on HLB and air permeability tests. The permeability of PBS through the PHB/Hmi membranes was studied, as PBS is a model substance which is similar to the environment of a human body [57]. Permeability of PBS through the PHB/Hmi membranes gradually increased with increasing the content of PBS. When the content of Hmi was 5 wt.%, PBS permeability increased by a factor of 8–12. Hence, the modification of the PHB nonwovens with Hemin appeared to be a powerful tool that allowed the improvement in their performance as membranes and filters [45]. This improvement was provided both by the formation of a uniform fibrous network structure composed of thin fibers upon ES of the PHB/Hmi materials and their controlled HLB and wettability.

Evident disadvantages of all nonwoven membrane materials and filters are concerned with their poor mechanical characteristics [58]. However, the addition of even minor amounts of Hemin to PHB provided positive changes in the mechanical characteristics of the PHB/Hmi materials. Information on the mechanical characteristics of the PHB/Hmi membranes (tensile strength and elongation at break) is presented in Table 3 [59,60]. Stress-strain curves are given in the Supplementary Materials (Figure S1).

As follows from Table 3, tensile strength increased by a factor of 3.2, and the elongation at break increased by a factor of 1.7. This evidence can be explained by a better organization and a more uniform supramolecular structure of the nonwoven PHB/Hmi materials. Hence, the introduction of Hemin provides improved mechanical properties of the modified materials and this advantage seems to be of particular importance for their practical use as membranes and filters [59].

## 4. Conclusions

The modification and functionalization of electrospun PHB-based fibrous nonwoven membrane materials by the addition of a natural functional iron-containing additive Hemin offer a facile and versatile approach for the preparation of “all-green” innovative membrane materials with high performance and good mechanical characteristics. The presence of Hemin even in low concentrations in the feed polymer solution markedly contributes to the improvement of the electrospinning process in whole due to enhanced electrical conductivity, thus allowing preparation of fibrous materials with uniform structure, reduced density, controlled pore size distribution, and good performance. By varying the composition of the feed polymer solution and ES operating conditions, this approach provides a controlled structural design of fibrous membrane materials with desired functional properties. Additional benefits of this approach are concerned with an efficient functionalization of the as-prepared membrane materials as their HLB can be tailored, and hydrophilicity is improved. The PHB/Hmi materials showed high sorption capacity and can be recommended for their use as sorbents and comfort breathable materials for biomedical applications, including wound healing. High air and water permeability PHB/Hmi membrane materials open up new horizons for their use as filters and membranes for water recovery, oil-water separation, and biomedical applications, including tissue engineering. When pristine PHB nonwovens suffer from poor mechanical characteristics, improved mechanical properties of the PHB/Hmi materials are of primary importance for their practical applications as membranes and filters. Due to antibacterial activity of Hmi, the advanced PHB/Hmi materials can be recommended for their use as biomedical membrane materials (wound healing, facial masks, protective comfort clothing, biomedical and cosmetic textiles, barrier materials for air filtering, etc.). It is noteworthy that the proposed PHB/Hmi membrane materials contain only natural components: biodegradable PHB and natural Hemin. Hence, these materials can be rated as environmentally friendly and “all-green” membranes and all problems related to their disposal and utilization can be easily solved. This approach opens up new horizons for the preparation of new “green” high-performance membrane materials, and further work in this direction should be focused on the search for new polymers and functional additives and on the evaluation of the potential of the resultant functionalized membrane materials for their efficient use in industry and daily life.

## Figures and Tables

**Table 1:** Composition of feed solutions and morphology of the PHB/Hmi membrane materials.

Content of Hmi, wt.%	Electrical Conductivity, μS/cm	Viscosity, Pa s	Density, g/cm^3^ Δ ± 0.01 g/cm^3^	Average Diameter, µm Δ ± 0.04 µm
0	10	1.0	0.30	3.50
1	11	1.4	0.20	2.06
3	13	1.7	0.20	1.77
5	14	1.9	0.17	1.77

**Table 2:** Supramolecular and phase structure of electrospun membrane PHB/Hmi materials.

Content of Hmi, wt.%	Degree of Crystallinity of PHB, %	Dimensions of PHB Crystallites *L*_020_, nm	Melting Temperature, °C	Heat of Fusion, J/g
0	47.8	26.8	175	93.1
1	45.0	33.2	172	81.8
3	41.0	31.2	173	77.8
5	40.6	30.8	174	75.3

**Table 3:** Mechanical characteristics of electrospun PHB/Hmi membrane materials.

Content of Hmi, wt.%	Tensile Strength, MPa Δ ± 0.02 MPa	Elongation at Break, % Δ ± 0.2%
0	1.7	3.6
1	0.7	4.7
3	1.9	4.7
5	5.5	6.1

## Data Availability

Not applicable.

## Supplementary Materials

The following supporting information can be downloaded at: https://www.mdpi.com/article/10.3390/membranes13050478/s1, Figure S1: Mechanical tests curves of samples PHB-Hmi.
