# Supplementary material for: Structure and Performance of All-Green Electrospun PHB-Based Membrane Fibrous Biomaterials Modified with Hemin
Source: Membranes (Basel). 2023 Apr 28;13(5):478. doi: 10.3390/membranes13050478 (PMC10221260; doi:10.3390/membranes13050478)
Supplement: Supplementary file 1 [file membranes-13-00478-s001.zip › membranes-2354440-supplementary.pdf]

membranes MDPI

Supplementary Material

Structure and Performance of All-Green Electrospun PHB-Based Membrane Fibrous Biomaterials Modified with Hemin

Polina M. Tyubaeva [1,2], Ivetta A. Varyan [1,2], Alexey V. Krivandin [2], Olga V. Shatalova [2], Anatoly A. Olkhov [1,2], Anatoly A. Popov [1,2], Huaizhong Xu [3] and Olga V. Arzhakova [4,*]

[1] Academic Department of Innovational Materials and Technologies Chemistry, Plekhanov Russian University of Economics, 36 Stremyanny per., Moscow 117997, Russia
[2] Emanuel Institute of Biochemical Physics, Russian Academy of Sciences, 4 ul. Kosygina, Moscow 119334, Russia
[3] Department of Biobased Materials Science, Kyoto Institute of Technology, Kyoto 606-8585, Japan
[4] Faculty of Chemistry, Lomonosov Moscow State University, Leninskie Gory 1/3, Moscow 119991, Russia
* Correspondence: arzhakova8888@gmail.com

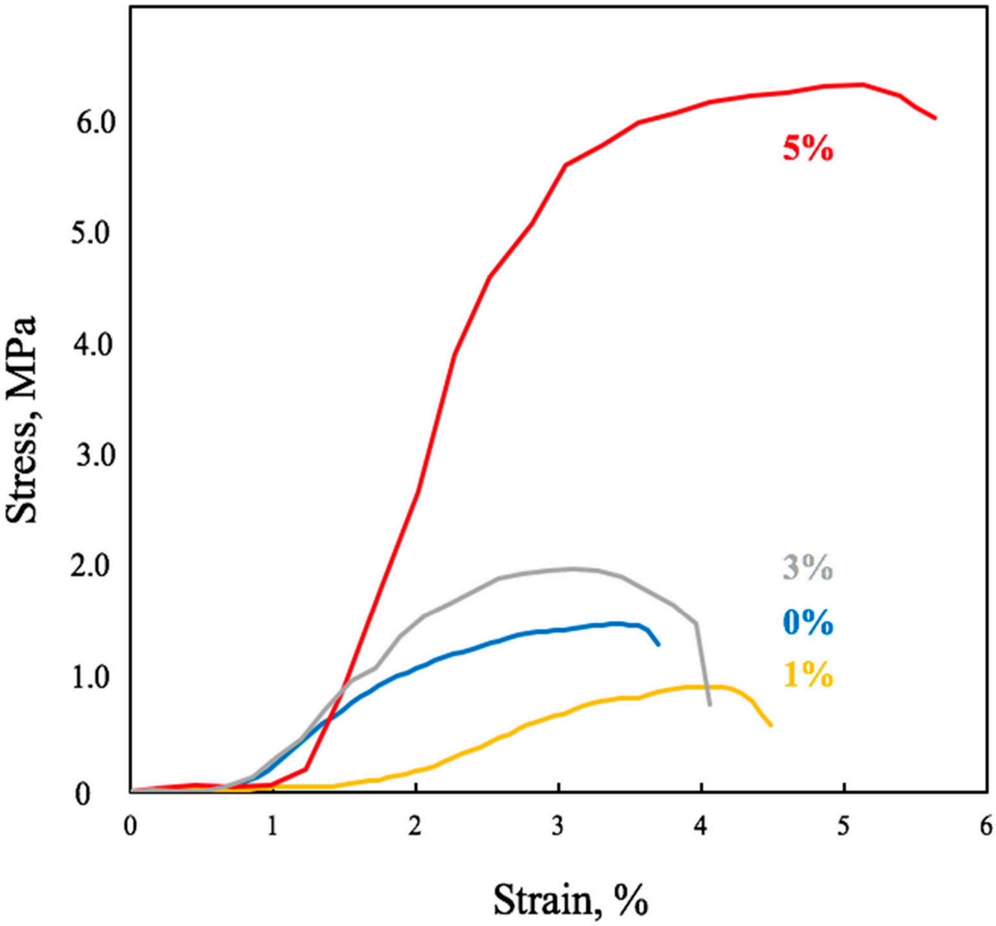

Figure S1. Mechanical tests curves of samples PHB-Hmi.
